# Iron-Containing Flocs Derived from Environmental Emergency Response Influenced Nitrogen Cycling Driven by Microorganisms in River Sediments

**DOI:** 10.3390/microorganisms14050980

**Published:** 2026-04-27

**Authors:** Zeqiang Huang, Sili Chen, An Fan, Yun Chen, Qijia Cai, Taotao Zeng, Weimin Zheng, Yuyin Yang

**Affiliations:** 1 School of Civil Engineering, University of South China, Hengyang 421001, China; aragorn0917@foxmail.com (Z.H.); 18838116373@163.com (A.F.); biowater@126.com (T.Z.); 2 Key Laboratory of Ecological Environment Emergency and Sudden Risk Control, South China Institute of Environmental Sciences (SCIES), Ministry of Ecology and Environment (MEE), Guangzhou 510655, China; chensili@scies.org (S.C.); chenyun@scies.org (Y.C.); caiqijia@scies.org (Q.C.)

**Keywords:** emergency response, nitrogen cycling, metal spill, river sediment, iron-containing flocs

## Abstract

In situ coagulation is regarded as the most effective measure in response to the frequent metal spills in China. Excessive coagulant is often used in pursuit of extremely high removal rates of contaminants. Yet the secondary ecological impact of the iron-containing coagulation flocs left on the river sediments after emergency response is still unclear. In the current study, we investigated the impact of flocs derived from three different iron-based coagulants, polymeric ferric sulfate (PFS), polymeric ferric chloride (PFC), and ferric chloride (FeCl_3_), on microbial communities in sediment based on microcosm experiments. Metagenomics, quantitative PCR, and determination of ammonia oxidation potential were adopted to elucidate community shifts. The results indicate that the community structure and function of microorganisms in sediments have been affected, especially processes and species related to nitrogen cycling, and the effect was coagulant-specific. Flocs retrieved from FeCl_3_ caused a more pronounced decline in diversity, shifts in community composition, and decreased potential ammonia oxidation. Ammonia-oxidizing archaea (AOA) was more sensitive to iron-containing flocs than ammonia-oxidizing bacteria (AOB), while PFS-flocs tended to reduce multiple genes involved in nitrate reduction. This indicates that the pre-polymerization of inorganic coagulants may be the primary factor leading to different microbial ecological effects. Sulfate, on the other hand, may affect specific biogeochemical processes due to its competition for electron donors. Our results confirmed that even without heavy metals as contaminants, coagulant flocs alone could present an effect on nitrogen cycling in sediments. The results will provide a scientific basis for environmental emergency decision-making: in emergency response to metal pollution incidents, the use of coagulants should be limited to only the necessary level.

## *1.* Introduction

Emergent water pollution incidents have occurred frequently in recent years and pose a serious threat to the environment and human health [1]. In emergency response to these incidents, the in situ coagulation–sedimentation method is widely used [2]. Coagulation has a high removal rate for oils and various heavy metals in water [3,4]. The low cost and ease of operation also help it become one of the most popular in situ emergency response measures [5]. However, due to the direct implementation of this technology in rivers, its potential ecological impact needs to be taken into consideration.

In in situ coagulation and sedimentation, ferric salts such as PFS (polymeric ferric sulfate), PFC (polymeric ferric chloride), and FeCl_3_ are among the most commonly used flocculants [6,7]. They are hydrolyzed in water to form iron hydroxide (Fe (OH)_3_) colloid, which can rapidly destabilize, aggregate and settle pollutants through mechanisms including compression of electric double layer, electrical neutralization, adsorption bridging and net capture and sweeping [8,9,10]. After the emergency response, the flocs are still deposited in the river channel, which may lead to a prolonged impact. On the one hand, the contaminants encapsulated within the flocs may slowly release to the water [11]. On the other hand, high levels of pollutants in sediment persist for years, posing long-term environmental stress on microorganisms [12]. Researchers noticed the enrichment of pollutants in sediments after environmental emergency response, and pointed out that this may lead to changes in sediment bacterial diversity and an increase in tolerant groups [13]. Metagenomic studies further confirmed functional changes in the cycling of elements such as carbon, nitrogen, and sulfur [14]. In addition, long-term stress from heavy metals may further select resistant microorganisms in sediments, resulting in co-selection of both heavy metal resistance and antibiotic resistance [15].

However, the long-term impact of in situ coagulation may not only come from pollutants such as heavy metals that co-precipitate in the flocs. Cai et al. [11] found that flocs generated by adding coagulant to clean river water without heavy metals may also lead to changes in the abundance of functional genes related to nitrogen and sulfur cycling. This may be related to the physical structure and chemical morphology of the flocs. Loose flocs precipitated on the sediment surface can serve as adsorbents and attachment matrices, promoting biofilm development and functional bacterial community enrichment [16,17]. When DWTR (drinking water treatment residue) with components similar to iron flocs is used as a suspended matrix in eutrophic water bodies, it can not only adsorb and immobilize excess phosphorus but also enrich a variety of nitrogen cycle-related microorganisms. The DWTR can possess almost a complete microbial nitrogen cycle pathway and exhibits a close co-occurrence relationship with iron-cycling microbes such as “*Ferrovum*” and *Geobacter*, while maintaining phosphorus adsorption capacity [18,19]. The iron hydroxide in the flocs can significantly increase the iron reserves in surface sediments, strengthening the coupling of iron redox and carbon–nitrogen–phosphorus cycles. The Fe(II) produced during the Fe(III) reduction process can abiotically consume nitrate/nitrite, supporting continuous nitrogen removal [20,21].

In emergency treatment, the ecological effects of coagulation residues can be understood as a linked sequence of processes. Coagulants are first added to rapidly destabilize and remove contaminants from the water column. This process generates iron-containing flocs, which subsequently settle onto the sediment surface. After deposition, these flocs may alter local physicochemical conditions by introducing reactive iron phases, associated anions such as sulfate or chloride, and additional surfaces for microbial colonization. Such changes may further influence microbial community structure and biogeochemical processes in sediments, particularly nitrogen cycling, which is closely linked to iron redox transformations in aquatic environments [22].

The three Iron-based coagulants examined in this study also differ in their chemical characteristics. PFS and PFC are pre-polymerized ferric coagulants that contain polymeric hydrolyzed Fe species before application, whereas FeCl_3_ is a monomeric ferric salt that mainly undergoes hydrolysis after addition to water [9,23,24]. These differences may affect floc formation, stability, particle structure, and surface reactivity, and may therefore contribute to differences in their ecological effects after sedimentation. In addition, the accompanying anions differ among coagulants, with sulfate introduced by PFS and chloride introduced by PFC and FeCl_3_, which may further influence specific biogeochemical processes.

In order to ensure a sufficiently pollutant removal rate of the contaminants, the coagulant is usually used at a high dosage [25], which may lead to massive deposition of iron in the river sediment. However, our understanding of the secondary ecological influence caused by excessive use of coagulants is still extremely limited. Very little is known about the differences in the effects of various coagulants.

In follow-up assessments of some pollution incidents, local authorities have reported downstream symptoms such as algal blooms and elevated ammonium concentrations in rivers or reservoirs, raising concerns that pollution itself or the associated emergency treatment processes may disturb nitrogen cycling. In addition, a previous microcosm study by Cai et al. further observed changes in the abundance of nitrogen-cycling-related genes in coagulant-amended treatments [11]. Because nitrogen cycling is one of the key biogeochemical processes in aquatic ecosystems, the present study focused particularly on microbially mediated nitrogen transformation.

In the current study, we explored the short-term effects of flocs derived from three widely used iron salt coagulants. Microcosms were constructed to simulate river sediments after floc sedimentation. We investigated the impact of iron-containing flocs on sediment environment and biological processes from the perspectives of physicochemical factors, microbial community structure, functional abundance and activity, with a special emphasis on nitrogen cycling. In particular, we examined whether these ecological effects were dependent on coagulant type and composition. The research results would enhance our understanding of the secondary ecological impacts of environmental emergency response and support a more scientific adaptation of in situ coagulation as an environmental emergency response technique.

## 2. Materials and Methods

### 2.1. Microcosms Setup

The environmental sample used for microcosm simulation was collected from a tributary upstream of Liuxi River National Forest Park. The sampling site was 23°44′41″ N, 113°47′11″ E. River sediment and the corresponding overlying water were collected according to the method described in the previous literature [26] and were transported to the laboratory on ice immediately.

The microcosmic experiment was used to simulate the effects of flocs from three different iron salt coagulant on the microbial community in the sediment–water system. To prepare iron-containing flocs, different iron salt coagulants (PFS (Shandong Xiya Chemical, Linyi, China), PFC (Fangxin Biotechnology, Guangzhou, China) and FeCl_3_ (Aladdin, Shanghai, China)) were prepared as 50 g/L stock and then added to the clean river water to reach an equivalent iron concentration of 5 g/L. The mixture was adjusted to pH 7.0–8.5, stirred at 200 rpm for 5 min and 40 rpm for 15 min, and then let stand to form flocs. A total of 50 g of sediment, 100 mL of river water and 10 g of iron-containing flocs were added to a 500 mL conical flask. 1 mL of 200 mg/L NH_4_Cl (Aladdin, Shanghai, China) was added to the mixture to provide additional ammonium substrate. In the blank control (BLK) group, the iron-containing flocs were replaced with an equal volume of clean river water, and the other treatments were the same as those in the experimental group. All of the treatments were carried out in triplicate and were incubated at 25 °C and 120 rpm for 7 days.

### 2.2. Physico-Chemical Analysis

After the 7-day incubation, the flasks were briefly let stand to allow the slurry to settle. A portable water quality analyzer (DZB-718L, Shanghai INESA Scientific Instrument, Shanghai, China) to determine the pH value and ORP (redox potential) of the supernatant. Then the supernatant was centrifuged at 7000 rpm for 5 min and filtered with a 0.22 μm filter. The physicochemical characteristics, including TN (total nitrogen), NH_4_^+^-N, NO_3_^−^-N and TOC were measured following the standard method as prescribed by the Ministry of Ecology and Environment of China [27]. To track the transfer of iron in different media, the concentrations of iron elements in the supernatant and sediment were measured, respectively. The concentration of iron in the supernatant was determined using phenanthroline spectrophotometry [28], while it in the sediment was determined using an ICP-MS (Inductively coupled plasma mass spectrometry) following the previously described procedure [29].

### 2.3. Determination of Potential Ammonia Oxidation

After the 7-day’ incubation, 5 g of sediment were collected from the microcosms and added to 20 mL of test medium, which contained 1 mM of phosphate, 15 mM of NaClO_3_ (Aladdin, Shanghai, China) and 1.5 mM (NH_4_)_2_SO_4_ (Aladdin, Shanghai, China). The mixture was incubated in a 100 mL flask at 25 °C and 250 rpm. The flask was shaken vigorously, and 2 mL of slurry was collected after 2 h and 6 h, respectively. 2 mL of 1 mM KCl (Aladdin, Shanghai, China) solution was added to the slurry to terminate ammonium oxidation and extract the nitrite in the slurry. Then the sample was centrifuged and filtered, and the concentration of the nitrite in the supernatant was determined by the spectrophotometric method. The ammonia oxidation potential (AOP) was calculated according to the linear growth rate of nitrite concentration in unit dry weight sediment within 2 to 6 h, given in mg NO_2_^−^N (kg dry sediment)^−1^ h^−1^. The dry weight of sediment was determined by drying and weighing the samples.

### 2.4. Molecular Analysis

On day 0 and day 7 of microcosm incubation, approximately 0.5 g sediment was collected from each flask. Genomic DNA was extracted from the sediment samples using a TIANamp Soil DNA Kit (Tiangen Biotech; Beijing, China). The integrity and purity of nucleic acid were examined on 1.5% agarose gel. And the concentration of DNA was measured using Nanodrop One photometer (Thermo Fisher Scientific; Waltham, MA, USA). The DNA samples were subjected to shotgun sequencing on Illumina NovaSeq 6000 PE150 platform (Illumina, San Diego, CA, USA) at Guangzhou Magigen Biotechnology Company (Guangzhou, China).

The raw reads were quality controlled using fastp (v0.23.2), and only reads with high quality were used for subsequent taxonomic classification and function annotation. Kraken2 (v2.1.3) and Bracken (v2.8) were adopted to assign taxonomy to the sequences according to GTDB (Genome Taxonomy Database, version2.3.2). Function annotation was carried out at the read level using HUMAnN3 (v3.0.1) with default parameters unless otherwise stated. Taxonomic profiling was first conducted using MetaPhlAn 3, and reads were then aligned against the UniRef90 database (release January 2019) using DIAMOND (v2.1.19). Gene family abundance tables and MetaCyc pathway abundance tables were generated for downstream analyses. Nitrogen-cycling genes analyzed in this study were extracted from the HUMAnN3 gene family output. The analyzed genes covered six major pathways, including nitrification, assimilatory nitrate reduction, dissimilatory nitrate reduction, denitrification, and nitrogen fixation. The complete list of genes used in downstream analysis is provided in Table S2. The raw sequence data reported in this paper have been deposited in the Genome Sequence Archive [30] at National Genomics Data Center [31], China National Center for Bioinformation, Chinese Academy of Sciences (GSA: CRA031940) that are publicly accessible at https://ngdc.cncb.ac.cn/gsa (accessed on 18 March 2026).

### 2.5. Quantification of Archaeal and Bacterial amoA Genes

Real-time quantitative PCR (qPCR) was performed to amplify the *amoA* genes of ammonia-oxidizing archaea (AOA) and ammonia-oxidizing bacteria (AOB). Archaeal *amoA* was amplified using the primer pair Arch-amoAF (5′-STAATGGTCTGGCTTAGACG-3′) and Arch-amoAR (5′-GCGGCCATCCATCTGTATGT-3′) [32], while bacterial *amoA* was amplified using the primer pair amoA-1F (5′-GGGGTTTCTACTGGTGGT-3′) and amoA-2R (5′-CCCCTCKGSAAAGCCTTCTTC-3′) [33].

qPCR assays were performed on a LightCycler 480 real-time PCR system (Roche, Basel, Switzerland). Each reaction was carried out in a total volume of 20 μL, containing 10 μL of 2× SYBR Green qPCR Master Mix (Tiangen Biotech, Beijing, China), 0.6 μL of each primer (10 μM), 5 μL of template DNA, and nuclease-free water to volume. All samples were analyzed in triplicate, and no-template controls (NTCs) were included in each run.

The amplification program consisted of an initial denaturation at 95 °C for 3 min, followed by 40 cycles of 95 °C for 15 s and 55 °C for 30 s. Melt curve analysis was performed after amplification to verify product specificity. Standard curves were generated using 10-fold serial dilutions (10^−2^ to 10^−8^) of plasmids carrying the target genes. The coefficients of determination (R^2^) of the standard curves were all greater than 0.99. The amplification efficiencies were 91.93% for archaeal *amoA* and 90.04% for bacterial *amoA*. Gene copy numbers were calculated based on the standard curves and expressed as copies g^−1^ wet sediment.

### 2.6. Statistics and Visualization

The statistical analysis and graphical visualization work were conducted using R software v4.5.0 [34]. The statistical significance of the experimental results was assessed in R (v4.5.0). A one-way ANOVA with a Tukey HSD post hoc test was used for multiple group comparisons, and *t*-tests were used for two-group comparisons. The microeco package (v1.16.0) [35] was used to analyze the sediment microbial community’s alpha diversity by calculating the Observed_OTUs, Chao1, Shannon and Simpson indices.

Principal coordinate analysis (PCoA) was used to visualize the differences in community structure among samples, and the significance of the differences among each treatment group was tested by PERMANOVA [36]. Differential abundance analysis was performed on microbial taxonomic units across different treatment groups, with the LEfSe (linear discriminant analysis effect size) method employed to identify significantly differentially abundant taxa [37,38]. The Pearson correlation coefficient matrix and corresponding significance *p*-values were calculated using the Hmisc package (v5.2.3) [39]. Furthermore, an environmental–microbial co-occurrence network was constructed based on these correlations. Network construction and topological metric calculations were performed using the igraph package (v2.1.4) [40], with network visualization achieved through the ggraph package [41]. The remaining results were visualized using the R packages ggplot2 (v3.5.2) [42] and pheatmap (v1.0.13) [43].

## 3. Results

### 3.1. Shifts in the Physico-Chemical Parameters Before and After the Incubation

**Figure 1:**
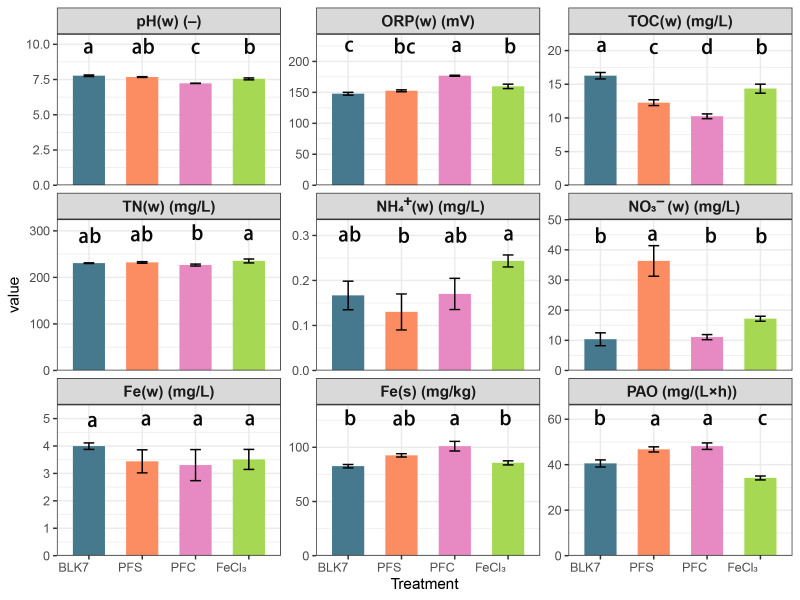
Effects of iron-containing floc treatment on overlying water and sediment physicochemical characteristics and sediment ammonium oxidation potential (PAO). Overlying water and sediment are labeled in parentheses and denoted by ‘w’ and ‘s’, respectively. Error bars indicate standard errors (n = 3). Different letters indicate statistically significant differences (*p* < 0.05).

After 7 days of incubation, microcosms amended with iron-containing flocs showed differences from the blank control (BLK7) in multiple physicochemical indicators (Figure 1). For the PFC treatment and the FeCl_3_ treatment, pH decreased slightly compared with the blank control (*p* < 0.05), while ORP increased significantly (*p* < 0.05). All kinds of flocs led to a decrease in the TOC of the overlying water (*p* < 0.05).

During the incubation, the concentration of NH_4_^+^-N in the overlying water decreased from 1.72 mg/L to 0.13–0.24 mg/L, while NO_3_^−^N increased from 7.40 to 10.34–36.32 mg/L. The concentration of NH_4_^+^-N in FeCl_3_ treatment was higher than that in PFS treatment, but no significant difference was observed between either treatment and the blank control. A remarkable higher concentration of NO_3_^−^N could also be identified in treatment PFS (*p* < 0.05), indicating a more active transformation of ammonia to nitrate. Further potential ammonia oxidation tests indicated that the amendment of PFS- and PFC-flocs led to more active nitrification in the sediment, while the addition of FeCl_3_-flocs had an opposite effect.

### 3.2. Structure of Sediment Microbial Community Amended with Fe-Containing Flocs

**Figure 2:**
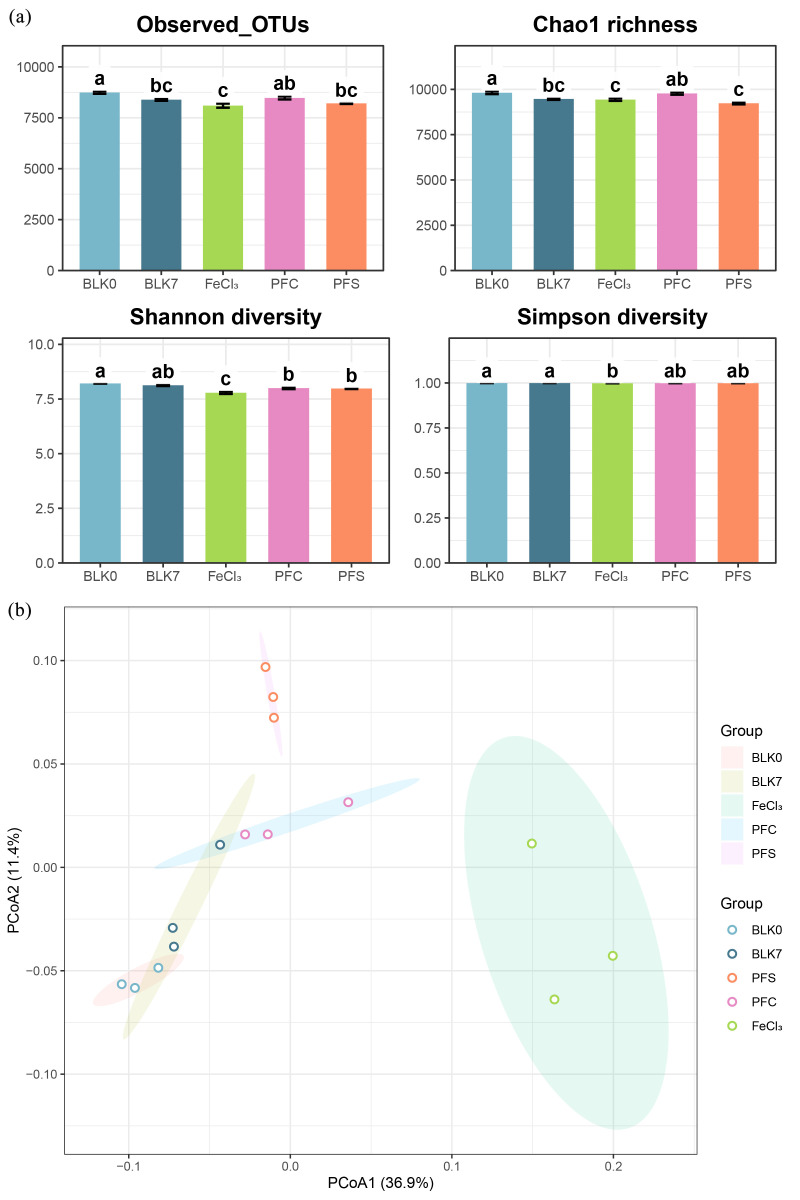
Effects of different iron-containing flocculant treatments on sediment microbial community α diversity and structure. (**a**) α diversity indices; different letters indicate significant differences (*p* < 0.05). (**b**) PCoA (Bray–Curtis distance); colors denote treatment groups, and points represent biological replicates.

The shift in microbial community diversity before and after cultivation were evaluated using several alpha diversity indices (Figure 2a). In the blank control group (BLK0 and BLK7), the overall α-diversity of the communities remained stable, suggesting that the cultivation process itself did not result in any notable changes to species richness or diversity. The flocs from FeCl_3_ coagulant led to a more notable decline in community diversity and richness than the other treatments. Principal coordinates analysis (PcoA) based on Bray–Curtis dissimilarity indicated remarkable a difference in microbial community structure among treatments (Figure 2b), as was also confirmed by PERMANOVA (*p* = 0.001). Samples from treatment FeCl_3_ were apart from the others, suggesting a notable impact of the flocs.

**Figure 3:**
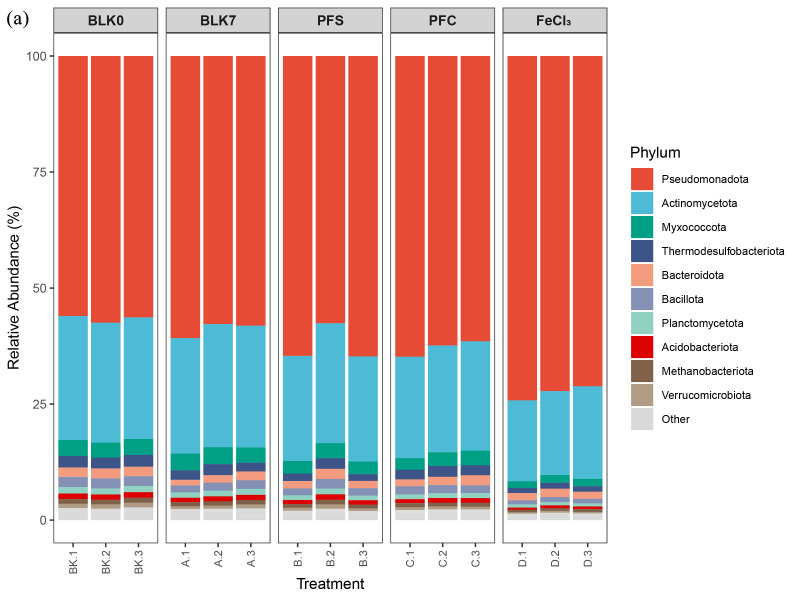

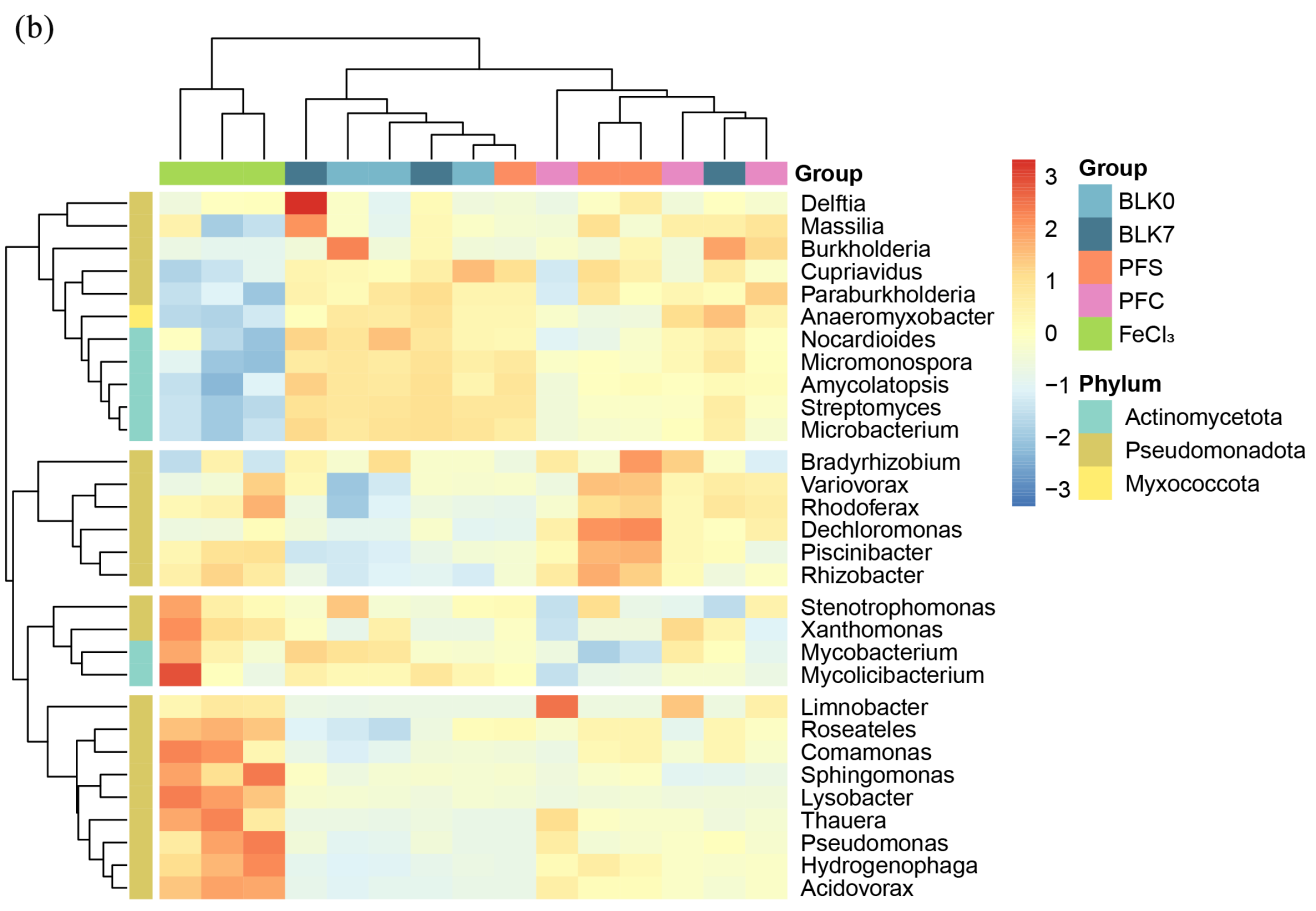

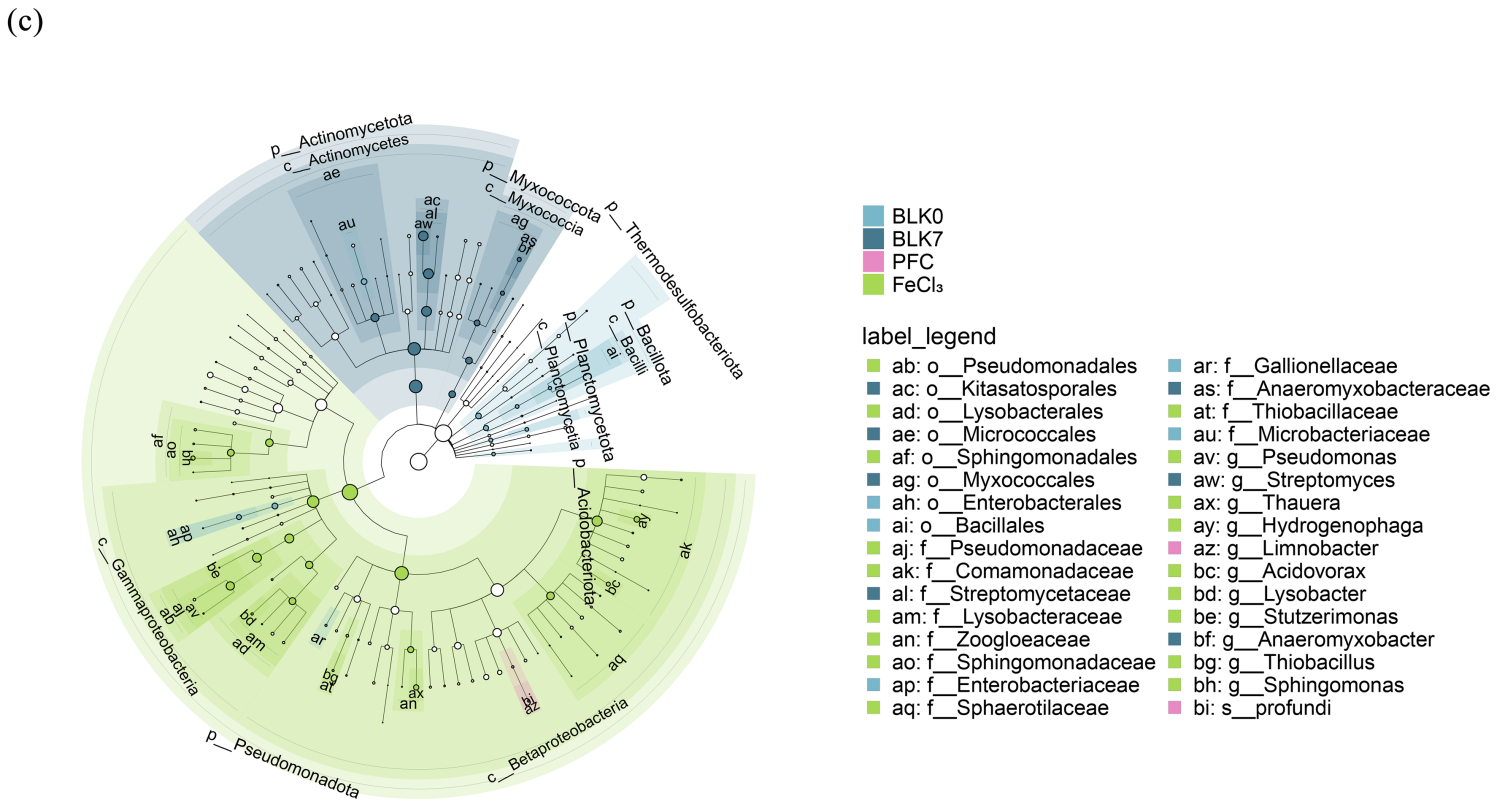
Bacterial community composition and differentially enriched taxa among different treatment groups. (**a**) Top 10 bacterial phyla by relative abundance. (**b**) Heatmap of the top 30 genera by relative abundance (>0.1%, Z-score). (**c**) LEfSe cladogram showing significantly enriched taxa.

Approximately 95.8% of the reads were annotated to the Bacterial, while 3.95% were related to Archaea. A total of 60 phyla were retrieved from the sequences, and the ten most abundant ones compromised 97.41–98.54% of the total community (Figure 3a). Pseudomonadota (previously known as Proteobacteria) was the most abundant phylum, with a relative abundance of 56.61–72.52% across the samples. It was enriched in microcosms amended with iron-containing flocs (62.31–72.52%), especially in the FeCl_3_ treatment (72.16–74.20%). Nitrospirota, which involved in nitrite oxidation, had a lower relative abundance in all the treatment groups (0.12–0.21%) compared with that in blank control (0.22–0.29%).

At genus level, 161 out of the 2504 annotated genera had an average relative abundance over 0.1%. Most of the abundant genera were affiliated with Pseudomonadota and Actinomycetota. Most genera from Acidobacteria were highly abundant in the blank control group (BLK0 and BLK7). On the other hand, genera affiliated with Pseudomonadota phylum were enriched in different treatments. Based on the genus-level composition, the samples clustered into three branches (Figure 3b). The FeCl_3_-floc-treated samples clustered into a separated one, while the other samples were roughly grouped into two clusters according to whether flocs were added.

Linear Discriminant Analysis Effect Size (LEfSe) analysis was performed at the genus level and above to identify microbial taxa exhibiting significant differences between treatment groups [44]. The results are presented in Figure 3c. Significant biomarker differences existed between groups (LDA score > 3.0, *p* < 0.05), indicating that the addition of iron flocs markedly altered the composition of the microbial community in the sediment.

Taxa that were enriched in the FeCl_3_-treated group primarily included Gammaproteobacteria, Burkholderiales, *Pseudomonas*, and *Thauera* within the Pseudomonadota phylum (Figure 3c). These genera are known to include members involved in organic matter degradation and nitrogen transformation. Their enrichment in the FeCl_3_-treated group suggests a shift toward taxa with greater potential for nitrogen redox processes. In contrast, the PFC group primarily enriched *Limnobacter* and the species-level *Limnobacter profundi*. The enriched communities in the control groups without flocculants (BLK0/BLK7) mainly comprised phyla related to Actinomycetota and Myxococcota, while BLK0 enriched Enterobacterales alongside Bacillota and Thermodesulfobacteriota. Overall, these results suggest that various treatments (particularly FeCl_3_) significantly drove sediment community differentiation and the selective enrichment of key groups.

### 3.3. Microbial Function Shifts After Amended with Iron-Containing Flocs

**Figure 4:**
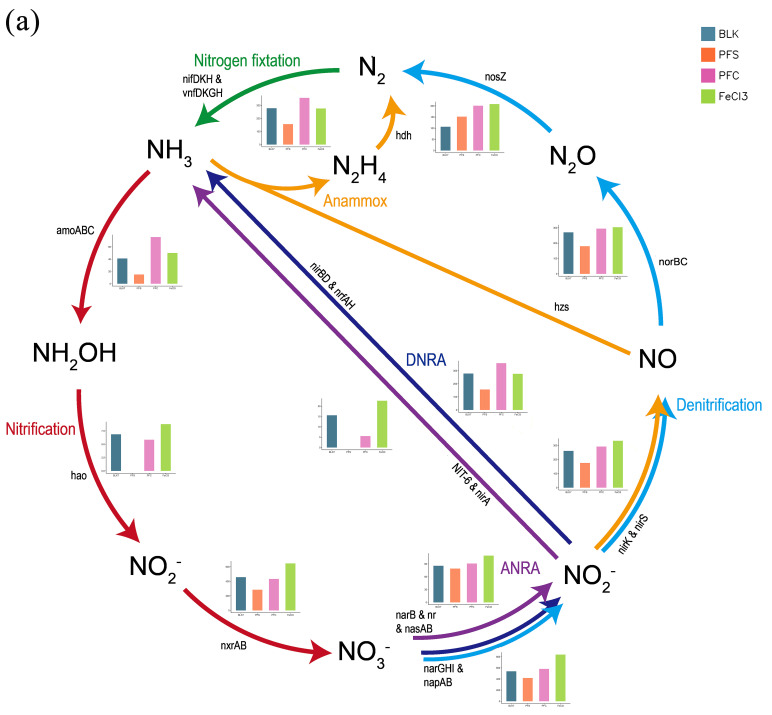

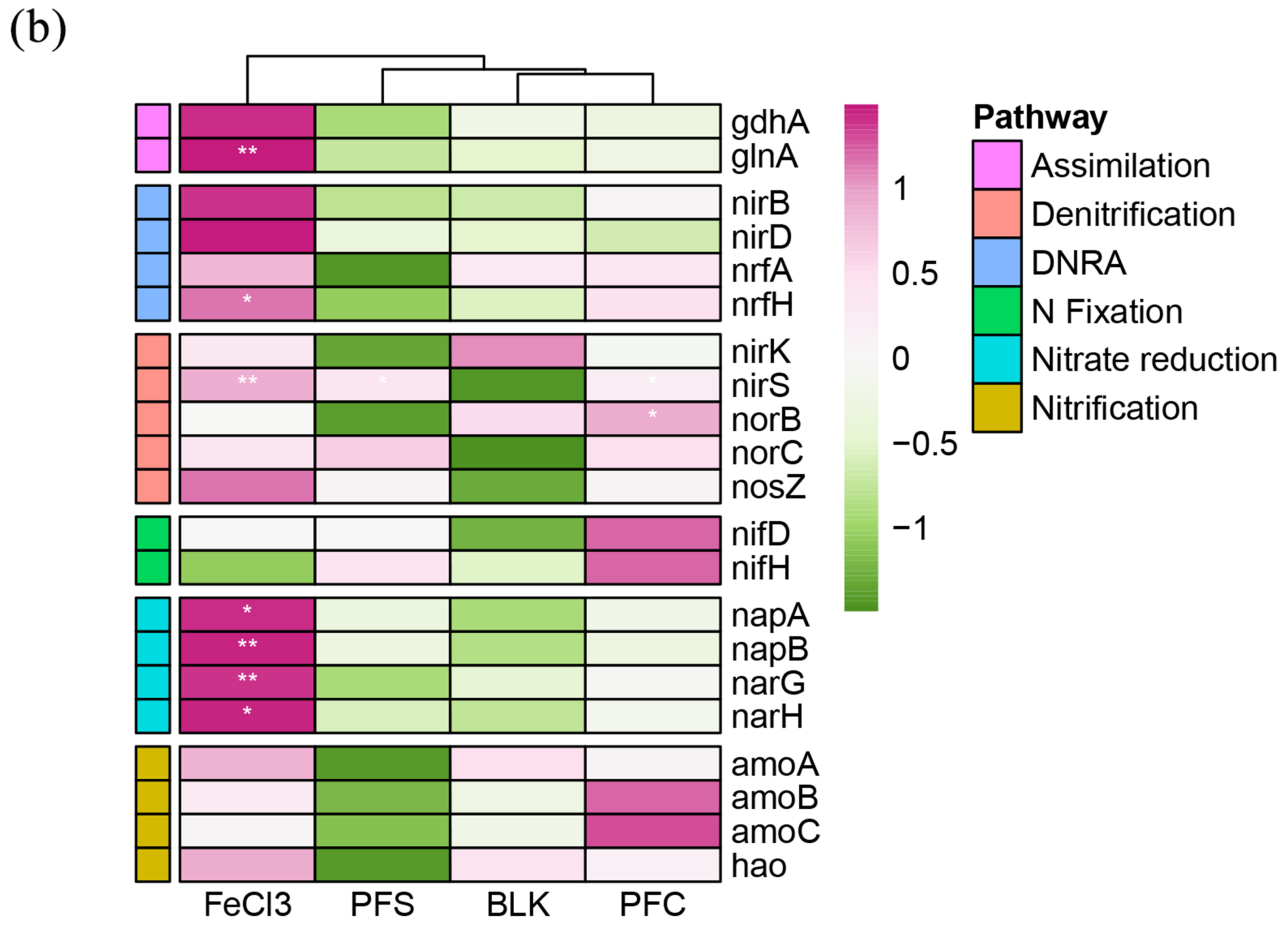
Effects of iron floc treatment on sediment nitrogen cycling functions. (**a**) Schematic diagram of nitrogen cycling pathway changes. (**b**) Heatmap of relative abundance of nitrogen cycling-related functional genes (Z-score). Significance was assessed using *t*-tests (* *p* < 0.05, ** *p* < 0.01).

An overall shift in the pathways related to the nitrogen cycle was shown in Figure 4a. For most of the pathways, PFC-floc-treated samples had a similar pathway abundance with that of FeCl_3_-floc-treated samples, while the PFS-floc-treated ones had the lowest pathway abundance. The decline in pathway abundance in the PFS group was particularly evident at the initial stage of the nitrification process, i.e., ammonia to hydroxylamine and hydroxylamine to nitrite.

The relative abundance of 21 genes related to 6 nitrogen cycling pathways were examined, and 7 genes presented significant differences among treatments (Figure 4b). Dissimilatory nitrate reduction seemed to be the most affected pathway, with most of the related genes encountering remarkable shifts after iron-containing flocs amendment. In general, they were favored by FeCl_3_- and PFC-flocs and inhibited by PFS-flocs. The relative abundance of most denitrification genes did not show significant differences among treatments.

Considering that different groups of bacteria and archaea were involved in the ammonia oxidation process, the qPCR of archaeal and bacterial *amoA* gene were carried out as a Supplement (Figure S1). Results indicated that in all the samples, more *amoA* genes were from the archaeal communities. After 7 days of incubation, the proportion of archaea AOA further increased. However, the *amoA* gene derived from archaea is more sensitive to the addition of iron-containing flocs. In the treatment group with Fe-flocs, the gene abundance of archaeal *amoA* decreased 33.12–44.58% compared with the control group, while there was no significant change in the bacterial *amoA*.

### 3.4. The Correlation Between Microbial Communities and Environmental Factors

**Figure 5:**
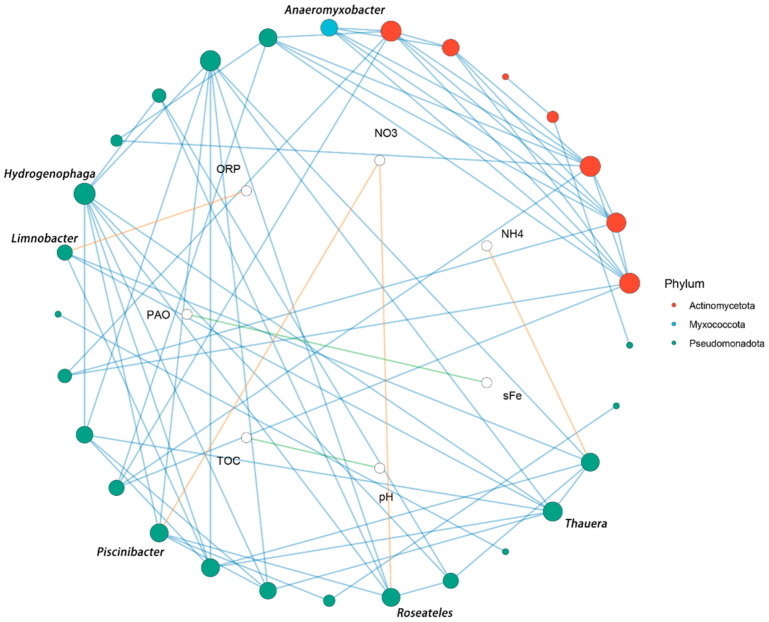
Correlation network between genus-level taxa and environmental factors.

In order to provide further elucidation on the regulatory role of environmental factors on microbial communities in sediments treated with iron-containing flocculants, this study constructed a network based on Spearman’s correlation coefficients between environmental factors and genus-level relative abundances. It is evident that only those correlations that were significantly positive correlations (*p* < 0.05, r > 0.7) were retained as network connections (Figure 5). The network comprised 36 nodes and 71 edges, with a network density of 0.113 and an average degree of 3.94, indicating moderate overall connectivity. The global clustering coefficient of 0.622 indicated the presence of distinct clustering among the nodes. Louvain community partitioning yielded seven modules with a modularity of 0.571, suggesting pronounced modularity in the network. The classification of genus nodes by phylum revealed Pseudomonadota as the most abundant phylum (21 nodes), followed by Actinomycetota (7 nodes) and Myxococcota (1 node). With regard to the analysis of node connectivity, it was observed that although *Pseudomonadota* contained the most nodes, *Actinomycetota* exhibited a higher average connectivity (5.57). This finding suggests that actinomycete-related genera contribute more densely within the network. In order to identify the key nodes, the following metrics were calculated degree, betweenness centrality, closeness centrality, and eigenvector centrality. Ranked by degree value, the genus with the highest degree value in the network was *Hydrogenophaga* (degree = 9). Among physicochemical factor nodes, the factor with the highest connectivity, as determined by degree value, was NO_3_^−^ (degree = 2). Furthermore, the genus-level environmental factor association statistics revealed that Pseudomonadota exhibited the highest number of significant correlation edges with environmental factors (n = 4). This finding suggests that, at the level of the threshold employed in this study, environmental factors such as nitrogen form, redox conditions, and soluble iron exhibit a stronger association with the Pseudomonadota-related group.

## 4. Discussion

### 4.1. Difference Among the Effect of Flocs from Various Iron-Based Coagulants

In this study, the effects of flocs generated from three different iron-based coagulants on sediment microbial communities were tested. Overall, the community composition underwent changes compared to the blank control. This might be related to the organic carbon transfer caused by the coagulant flocs. In all treatments, TOC in the overlying water decreased significantly (Figure 1). Although the formation of the coagulant flocs did not occur in the microcosms, the directly added flocs could still adsorb organic substrate from the water and transfer it to the sediment. This might provide the microorganisms with more abundant substrates, resulting in changes in community structure.

Among different coagulants, flocs derived from ferric chloride caused more prominent effects on the sediment microbial community. For example, a significant decrease in diversity and richness (Figure 2a) and notable structural shifts in the community (Figure 2b) were observed in the ferric chloride floc treatment. LEfSe also confirmed multiple microbial taxa enriched (Figure 3c). Polymerized iron salts and monomeric iron salts exhibited divergent impacts on the microbial community, whereas different anions only presented a minor influence. This might be related to the differences in the hydrolysis forms of Fe(III) formed by polymeric and monomeric iron salts during the coagulation process, as well as the differences in floc structure [9].

Fe(III) existed in various forms upon the hydrolysis of iron salts, including monomeric hydroxyl complexes, polymeric hydroxyl-iron species, and further formed Fe(III)(hydr)oxides colloids or precipitates [45]. PFS and PFC typically contained a certain proportion of pre-polymerized iron [23,24], generating abundant polymeric hydroxyl-iron species. In contrast, FeCl_3_ relied more on immediate hydrolysis and polymerization processes after addition. The composition of various iron species influenced the formation pathways of flocs, leading to variations in particle size, compactness, and pore structure, thereby altering the surface sites and reactivity of the flocs [9]. Shi et al. [46] pointed out that PFS exhibited lower corrosivity and fewer iron residues compared to FeCl_3_, with Fe (III) tending to settle in a stable flocculated form. Dong et al. [47] also confirmed that Fe (III) species composition and floc properties differ between the flocs derived from FeCl_3_ and PFC, and polymeric iron salts were more likely to form stable particulate iron phases and reduce disturbances in the aqueous phase. Therefore, flocs derived from FeCl_3_ might be less stable, thus exerting a more pronounced impact on microbial communities. This was also supported by the lower iron concentration in sediment observed in the FeCl_3_-floc treatment in this study (Figure 1). Furthermore, the chlorine-containing ferric hydroxide formed through the hydroxylation of iron with Cl^−^ could further alter the iron phase structure and surface properties [48], thereby widening the community differences among treatments.

### 4.2. Nitrogen Cycling Affected by Iron-Containing Flocs

In aquatic environments, the nitrogen cycle and iron cycle driven by microorganisms are tightly coupled [49]. Therefore, it is necessary to demonstrate the potential impact of the extensive sedimentation of iron-containing coagulated flocs on the nitrogen cycle. In the initial stage of aerobic nitrogen transformation, we investigated the effect of iron-containing flocs on ammonia oxidation from the perspectives of reaction potential (Figure 1), functional gene quantification, and metagenomic, and the trend were not exactly the same.

The determination of PAO provided indicated that the oxidation of ammonium could be promoted by PFS- and PFC-flocs, but inhibited by FeCl_3_-flocs, under aerobic conditions with sufficient ammonium. The correlation between PAO differences and *amoA* abundance was insignificant, which was similar to that in other iron-rich systems [50,51]. The qPCR results indicated that AOA predominated in the ammonia-oxidizing community within the environmental samples used in this study, exhibiting a relative abundance approximately one order of magnitude higher than that of AOB. However, AOA showed greater sensitivity to the amendment of flocs, with a significant reduction in all treatments. In natural environments, AOA and AOB occupy distinct ecological niches. AOA are generally more adapted to low-ammonia and relatively stable conditions, whereas AOB tend to become dominant under higher substrate concentrations or more disturbed environments [52]. Consequently, the sensitivity of the ammonia-oxidizing community and its associated processes to iron-containing flocs may be governed by the initial community structure. On the other hand, during the measurement of PAO, the effectiveness of chlorate in inhibiting the nitrification process might have been interfered with by Comammox bacteria [53]. From our metagenomic results, we annotated *Candidatus Nitrospira inopinata*, a model species of Comammox [54], whose relative abundance also significantly decreased in the FeCl_3_-floc treatment (Figures S2 and S3). This supported the inhibitory effect of FeCl_3_-floc treatment on ammonia transformation processes.

Flocs and the low shaking speeds might create a localized hypoxic condition, thereby allowing different nitrogen transformation processes to occur within the microcosms. The relative enrichment of some facultative anaerobic microorganisms also supported this inference. In the PFS-floc treatment, nitrate accumulated in the slurries (Figure 1). The accumulation of downstream products might be the reason of the downregulation of functional genes related to nitrification (Figure 4). During the hydrolysis and sedimentation of PFS, SO_4_^2−^ could be encapsulated within the porous structure of the flocs and subsequently released into the aqueous phase during agitation [23]. In natural freshwater ecosystems, sulfate usually remained at a low level [55]. The sulfate introduced by the flocs might compete with nitrate for electron donors, thus reducing nitrate conversion in the PFS-floc treatment.

### 4.3. Microbial Taxa Favored by FeCl_3_-Flocs

In the FC treatment group, Pseudomonadota exhibited a significantly higher relative abundance compared to other treatments (Figure 2a). Pseudomonadota is a commonly dominant phylum in river sediments with relatively high tolerance to pollutant stress [56,57]. Its enrichment suggested that the amendment of FC-flocs might have imposed stronger environmental pressure on the sediment microbial community compared to polymeric coagulants. LEfSe results indicated that the FC treatment showed a preference for taxa capable of facultative heterotrophy and possessing metabolic genes associated with nitrate respiration, such as *Thauera* and *Hydrogenophaga* [58,59]. *Thauera* was previously reported as a dominant denitrifying group in various wastewater biological treatment systems and frequently linked to NO_3_^−^/NO_2_^−^ reduction processes [60]. Isolates of *Hydrogenophaga* was identified with heterotrophic nitrification–aerobic denitrification capabilities [58]. The high connectivity observed in the ecological network suggested that *Thauera* and *Hydrogenophaga* likely occupied important positions in community interactions. Their enrichment in the FC treatment might have contributed to maintaining the relative stability of community structure and function under environmental stress [61,62].

Iron-cycling related taxa, such as *Shewanella oneidensis*, were also enriched in the FeCl_3_ treatment (Table S1). *S. oneidensis* is a model bacterium for dissimilatory metal reduction and extracellular electron transfer (EET), capable of transferring electrons to insoluble extracellular acceptors such as Fe(III) (hydr)oxides, thereby driving Fe(III) reduction [63]. This result suggested that FeCl_3_ treatment likely exerted a selective pressure on facultative microorganisms possessing metal-reducing capabilities by increasing the availability of Fe(III) in the system. Some iron-reducing microorganisms often possessed the metabolic potential to utilize nitrate as an alternative electron acceptor [64], which might explain the increased abundance of denitrification pathways according to metagenomic functional annotation.

In the present study, metagenomic results were interpreted based on relative abundance rather than absolute abundance. However, changes in relative abundance do not necessarily reflect proportional changes in the absolute population size of specific taxa or functional groups. We adopted qPCR of archaeal and bacterial amoA to provide quantitative information for ammonia oxidizers, yet absolute abundance data were not available for most microbial taxa and nitrogen-cycling genes. Therefore, the metagenomic results should be interpreted primarily as changes in community composition and functional potential. In addition, this study was based on a short-term aerobic microcosm experiment and does not fully represent natural river sediments. Future studies should include longer-term experiments, more realistic redox conditions, broader absolute quantification approaches, and methods such as metatranscriptomics and isotope-labeling techniques to better evaluate microbially mediated nitrogen transformation in river ecosystems.

## 5. Conclusions

We investigated the effects of flocs generated by coagulation as an emergency response strategy on sedimentary microorganisms and compared the differences among various iron-based coagulants. The results indicated that coagulation flocs might alter the structure and function of microbial communities in sediments, which is associated with the type of coagulant employed. Molecular weight was considered a primary influencing factor: non-polymerized ferric chloride caused a more pronounced decline in diversity, shifts in community composition, and decreased potential ammonia oxidation. Anions could influence specific biogeochemical processes, with sulfate-containing coagulants tending to affect nitrate transformation. The sensitivity of nitrogen cycling processes to iron salt flocs might be related to the initial community composition. The findings suggested that the large-scale application of coagulants during environmental emergency responses would impact riverine microbial ecology and biogeochemical processes. Therefore, when treatment performance allows, pre-polymerized iron coagulants may be preferable to ferric chloride. The dosage of coagulant should be controlled at the minimum amount required for contamination removal. And dredging should be carried out after in situ coagulation if possible. However, this study was based on a short-term aerobic microcosm experiment and does not fully represent natural river sediments. In natural rivers, hydraulic disturbance, fluctuating redox conditions, and spatial heterogeneity may make microbial responses to coagulation flocs more complex than those observed in a simple shaking-flask system. Future studies should therefore incorporate continuous-flow simulations or in situ river investigations to better evaluate the microbial ecological effects of coagulation flocs under realistic environmental conditions.

## Data Availability

The raw sequence data reported in this paper have been deposited in the Genome Sequence Archive at National Genomics Data Center, China National Center for Bioinformation, Chinese Academy of Sciences (GSA: CRA031940) that are publicly accessible at https://ngdc.cncb.ac.cn/gsa (accessed on 18 March 2026).

## Supplementary Materials

The following supporting information can be downloaded at: https://www.mdpi.com/article/10.3390/microorganisms14050980/s1, Figure S1: qPCR-determined copy numbers of the amoA gene encoding ammonia-oxidizing microorganisms in sediments treated with different iron-containing flocculants. (a) AOA-amoA; (b) AOB-amoA. Bars show mean ± SD (n = 3); different letters indicate significant differences (*p* < 0.05); Figure S2: Heatmap of nitrification-related genera across treatments. Colors indicate standardized relative abundance (Z-score). Different letters within a genus denote significant differences among treatment groups (*p* < 0.05); Figure S3: Relative abundance of *Candidatus Nitrospira inopinata* across treatments. Bars represent mean ± SD (n = 3). Different letters indicate significant differences among groups (*p* < 0.05); Table S1: Relative abundance of *Shewanella oneidensis* across treatments (biological replicates); Table S2: Nitrogen-cycling genes included in functional analysis.
