# Supplementary material for: Iron-Containing Flocs Derived from Environmental Emergency Response Influenced Nitrogen Cycling Driven by Microorganisms in River Sediments
Source: Microorganisms. 2026 Apr 27;14(5):980. doi: 10.3390/microorganisms14050980 (PMC13209269; doi:10.3390/microorganisms14050980)
Supplement: Supplementary file 1 [file microorganisms-14-00980-s001.zip › microorganisms-4240652-supplementary.pdf]

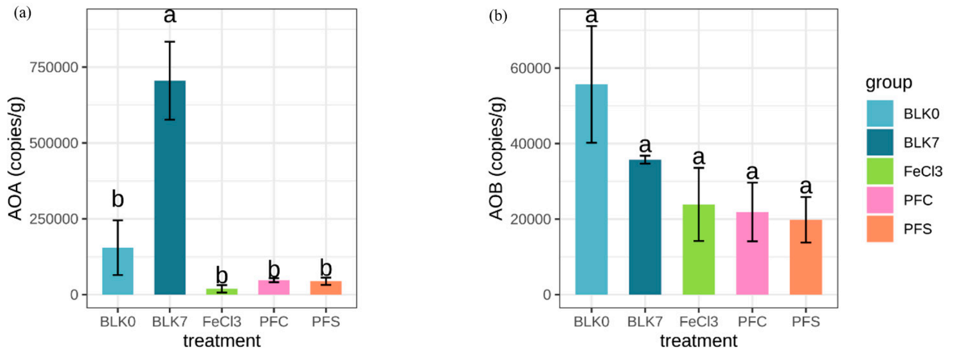

Figure S1. qPCR-determined copy numbers of the amoA gene encoding ammonia-oxidizing microorganisms in sediments treated with different iron-containing flocculants. (a) AOA-amoA; (b) AOB-amoA. Bars show mean ± SD (n = 3); different letters indicate significant differences ($p < 0.05$).

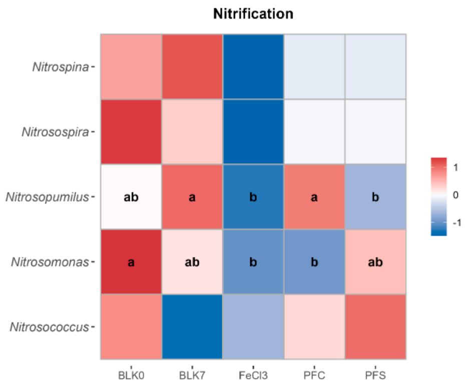

Figure S2. Heatmap of nitrification-related genera across treatments. Colors indicate standardized relative abundance (Z-score). Different letters within a genus denote significant differences among treatment groups ($p < 0.05$).

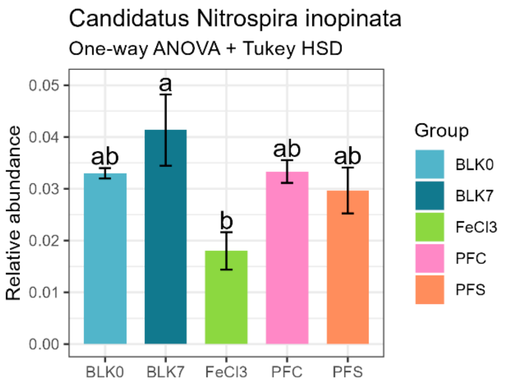

Figure S3. Relative abundance of *Candidatus Nitrospira inopinata* across treatments. Bars represent mean ± SD (n = 3). Different letters indicate significant differences among groups ($p < 0.05$).

Table S1. Relative abundance of Shewanella oneidensis across treatments (biological replicates).

Treatment	Relative abundance (mean ± SD, n=3)
BLK0	0.00000 ± 0.00000
BLK7	0.00000 ± 0.00000
PFS	0.00001 ± 0.00001
PFC	0.000007 ± 0.000012
$FeCl_3$	0.000033 ± 0.000015

Table S2. Nitrogen-cycling genes included in functional analysis.

KO	Gene name	Function	Category
K02588	nifH	N_2 fixation	step1_Nitrogen_fixation
K02586	nifD	N_2 fixation	step1_Nitrogen_fixation
K00376	nosZ	Nitrous oxide reduction	step7_Nitrous_oxide_reduction
K00371	narH	Nitrate reduction	step4_Nitrate_reduction
K00370	narG	Nitrate reduction	step4_Nitrate_reduction
K02591	nifK	N_2 fixation	step1_Nitrogen_fixation
K02305	norC	Nitric oxide reduction	step6_Nitric_oxide_reduction
K15864	nirS	Nitrite reduction	step5_Nitrite_reduction
K15876	nrfH	Nitrite reduction to ammonia	step8_Nitrite_ammonification
K10945	amoB	Ammonia oxidation	step2_Ammonia_oxidation
K04561	norB	Nitric oxide reduction	step6_Nitric_oxide_reduction
K10946	amoC	Ammonia oxidation	step2_Ammonia_oxidation
K10944	amoA	Ammonia oxidation	step2_Ammonia_oxidation
K07218	nosD	Nitrous oxide reduction	step7_Nitrous_oxide_reduction
K02568	napB	Nitrate reduction	step4_Nitrate_reduction
K02567	napA	Nitrate reduction	step4_Nitrate_reduction
K03385	nrfA	Nitrite reduction to ammonia	step8_Nitrite_ammonification
K04015	nrfD	Nitrite reduction to ammonia	step8_Nitrite_ammonification
K00368	nirK	Nitrite reduction	step5_Nitrite_reduction
K00362	nirB	Nitrite reduction to ammonia	step8_Nitrite_ammonification
K00363	nirD	Nitrite reduction to ammonia	step8_Nitrite_ammonification
